# Genotype-Phenotype Correlations in *CYP1B1*-Associated Primary Congenital Glaucoma Patients Representing Two Large Cohorts from India and Brazil

**DOI:** 10.1371/journal.pone.0127147

**Published:** 2015-05-15

**Authors:** Mônica Barbosa de Melo, Anil K. Mandal, Ivan M. Tavares, Mohammed Hasnat Ali, Meha Kabra, José Paulo Cabral de Vasconcellos, Sirisha Senthil, Juliana M. F. Sallum, Inderjeet Kaur, Alberto J. Betinjane, Christiane R. Moura, Jayter S. Paula, Karita A. Costa, Mansoor Sarfarazi, Mauricio Della Paolera, Simone Finzi, Victor E. F. Ferraz, Vital P. Costa, Rubens Belfort, Subhabrata Chakrabarti

**Affiliations:** 1 Center of Molecular Biology and Genetic Engineering, University of Campinas, Campinas, SP, Brazil; 2 Jasti V Ramanamma Childrens Eye Care Centre, L.V. Prasad Eye Institute, Hyderabad, India; 3 Department of Ophthalmology, Federal University of São Paulo, SP, Brazil; 4 Centre for Clinical Epidemiology and Biostatistics, L.V. Prasad Eye Institute, Hyderabad, India; 5 Kallam Anji Reddy Molecular Genetics Laboratory, L.V. Prasad Eye Institute, Hyderabad, India; 6 Department of Ophthalmology, Faculty of Medical Sciences, University of Campinas, Campinas, SP, Brazil; 7 Department of Ophthalmology, University of São Paulo School of Medicine, São Paulo, SP, Brazil; 8 Department of Ophthalmology, Ribeirão Preto Medical School, University of São Paulo, Ribeirão Preto, SP, Brazil; 9 Molecular Ophthalmic Genetics Laboratory, Surgical Research Center, Department of Surgery, University of Connecticut Health Center, Farmington, Connecticut, United States of America; 10 Department of Ophthalmology, Irmandade da Santa Casa de Misericordia de São Paulo, School of Medical Sciences, São Paulo, SP, Brazil; 11 Genetics Department, Ribeirão Preto Medical School, University of São Paulo, Ribeirão Preto, SP, Brazil; National Eye Institute, UNITED STATES

## Abstract

**Background:**

Primary congenital glaucoma (PCG), occurs due to the developmental defects in the trabecular meshwork and anterior chamber angle in children. PCG exhibits genetic heterogeneity and the *CYP1B1* gene has been widely implicated worldwide. Despite the diverse mutation spectra, the clinical implications of these mutations are yet unclear. The present study attempted to delineate the clinical profile of PCG in the background of *CYP1B1* mutations from a large cohort of 901 subjects from India (n=601) and Brazil (n=300).

**Methods:**

Genotype-phenotype correlations was undertaken on clinically well characterized PCG cases from India (n=301) and Brazil (n=150) to assess the contributions of *CYP1B1* mutation on a set of demographic and clinical parameters. The demographic (gender, and history of consanguinity) and quantitative clinical (presenting intraocular pressure [IOP] and corneal diameter [CD]) parameters were considered as binary and continuous variables, respectively, for PCG patients in the background of the overall mutation spectra and also with respect to the prevalent mutations in India (R368H) and Brazil (4340delG). All these variables were fitted in a multivariate logistic regression model using the Akaike Information Criterion (AIC) to estimate the adjusted odds ratio (OR) using the R software (version 2.14.1).

**Results:**

The overall mutation spectrum were similar across the Indian and Brazilian PCG cases, despite significantly higher number of homozygous mutations in the former (p=0.024) and compound heterozygous mutations in the later (p=0.012). A wide allelic heterogeneity was observed and only 6 mutations were infrequently shared between these two populations. The adjusted ORs for the binary (demographic) and continuous (clinical) variables did not indicate any susceptibility to the observed mutations (p>0.05).

**Conclusions:**

The present study demonstrated a lack of genotype-phenotype correlation of the demographic and clinical traits to *CYP1B1* mutations in PCG at presentation. However, the susceptibility of these mutations to the long-term progression of these traits are yet to be deciphered.

## Introduction

Primary congenital glaucoma (PCG) is an autosomal recessive disease that occurs due to developmental defects in the trabecular meshwork and anterior chamber angle resulting in a severe form of visual impairment in children. [1–5] Surgical management involving the reduction of intraocular pressure (IOP) is the only treatment modality and untreated cases may result in irreversible blindness. [4,6] PCG exhibits a high prevalence in populations where inbreeding and consanguinity are common such as 1 in 1250 to 1 in 2500 to 1 in 3300 among the Slovakian gypsies, Saudi Arabians and a Southern Indian population, respectively. [7–9] The prevalence is relatively lower in the developed world and ranges from 1 in 10,000–30,000 livebirths. [10–11]

Genetic heterogeneity is the hallmark of PCG and locus heterogeneity is evident across the four chromosomal regions (*GLC3A* [OMIM 231300], *GLC3B* [OMIM 600975], *GLC3C* [OMIM 613085], *GLC3D* [OMIM 613086]) mapped by linkage analysis. [12–15] The *GLC3A* and the *GLC3D* loci have been further characterized to harbor mutations in the *CYP1B1* (OMIM 601771) and *LTBP2* (OMIM 602091) genes, respectively. [16–19] While *CYP1B1* exhibits wide allelic heterogeneity across multiple populations,[20–35] the *LTBP2* is largely responsible for atypical forms of congenital glaucoma and its mutation spectrum across different populations is currently being determined. [36–39]

Despite the varying frequencies of *CYP1B1* mutations worldwide, it has been observed that there is a greater degree of sharing of similar mutations across multiple ethnicities. [29, 40] Earlier, we demonstrated that the *CYP1B1* mutations are geographically well structured based on their intragenic haplotypes, indicating founder effects and population movements. [40–41] While this provides an excellent opportunity to undertake extensive genotype-phenotype correlations to characterize specific clinical traits in the background of *CYP1B1* mutation spectrum, such efforts are rarely seen in PCG. Nevertheless, there are some studies that did attempt to associate the effect of certain mutant genotypes on the phenotypes. [39,42–47] However, a concerted effort to unravel the overall picture of genotype-phenotype correlations pertaining to *CYP1B1* mutations that would help in understanding the clinical course of the disease for a better prognosis, is still elusive.

The *CYP1B1* gene has been widely analyzed in the Indian [26,45] and the Brazilian [48–50] PCG patients earlier with varying degrees of severity. We had also demonstrated that certain *CYP1B1* mutations occurred on the same haplotype backgrounds in these two populations. [40] We now aimed to understand the genotype-phenotype correlations in *CYP1B1*-associated PCG cases in a large cohort of subjects (n = 901) representing clinically well-characterized PCG patients and ethnically matched normal controls across two different ethnicities from India and Brazil.

## Methods

### Enrolment of the cohort

The work was accomplished through a joint research funding under the initiatives of the Ministries of Science and Technology of the Governments of India (Department of Science and Technology) and Brazil (CNPq). A consortium was developed with 5 major institutes from São Paulo in Brazil (University of Campinas, Federal University of São Paulo, Universities of São Paulo at São Paulo city and Ribeirão Preto and the Santa Casa de Misericordia de São Paulo) and a tertiary eye care centre at Hyderabad in India (LV Prasad Eye Institute). The total cohort included 901 subjects comprising 451 PCG patients (India [n = 301] and Brazil [n = 150]) and 450 controls (India [n = 300]; Brazil [n = 150]). It also included subjects characterized earlier from India and Brazil along with the new cases. [45,48,50] A consensus was developed through joint meeting of investigators in both the countries and disease definitions and clinical criteria for enrolment were harmonized to facilitate genotype-phenotype correlation.

The inclusion criteria for patients were as defined earlier and those with secondary causes of disease were excluded. [40] Ethnically matched normal volunteers without any signs or symptoms of glaucoma or other ocular and systemic diseases were enrolled as controls. The enrolments of subjects were strictly monitored by clinicians across both the countries with expertise in congenital glaucoma diagnosis and treatment.

### Ethics statement

The study protocols adhered to the tenets of the Declaration of Helsinki and were approved by the Institutional Review Boards (IRB) at each of the 5 centres in Brazil and one from India. A written informed consent was obtained from each participant at all the study sites prior to their enrolment in the study. In case of minors, the written informed consent was obtained from their parents or legal guardians. The IRB approvals were obtained by each PI from their respective organizations, which are listed below:

- University of Campinas, Campinas, São Paulo, Brazil (IRB Approval No. 976/2009),
- Federal University of São Paulo, São Paulo, Brazil (IRB Approval No. 1376/09),
- University of São Paulo School of Medicine, São Paulo, Brazil (IRB Approval No. 753/06),
- Ribeirão Preto Medical School, University of São Paulo, São Paulo, Brazil (IRB Approval No. HCRP 14631/2009)
- Irmandade da Santa Casa de Misericordia de São Paulo, School of Medical Sciences (IRB Approval No. 178/04)
- L.V. Prasad Eye Institute, Hyderabad, India (IRB Approval No. LEC/08/2009)

### Clinical and demographic details of the subjects

The demographic details of the subjects including their gender, age at onset of disease symptoms, geographical region of origin, family history of glaucoma and consanguinity were noted. The "age at onset" was defined as the time since the signs and/or symptoms of PCG were first visible to the parent or legal guardian of the PCG-affected child. Additionally, all the new subjects underwent a comprehensive clinical examination and ocular parameters at presentation (intraocular pressure (IOP), corneal diameter, ratio of optic cup to optic disc, visual acuity and corneal clarity) similar to the existing cohorts.

### Molecular analysis

The two coding exons of *CYP1B1* gene were screened by resequencing in all the new subjects with appropriate primers on an automated DNA sequencer ABI 3130xl (Applied Biosystems, Foster City, CA) using the BigDye chemistry following the manufacturer’s guidelines. [40] A variation was termed as ‘mutation’ based on the criteria detailed earlier [40] and the SIFT analysis was also done to determine the pathogenic nature of the variant. Validation of an observed variant was further undertaken by resequencing using the above technique. Six intragenic polymorphisms in *CYP1B1* were used to generate haplotypes for cases with and without mutations and the normal controls. The estimated haplotype frequencies, measures of Hardy Weinberg equilibrium and linkage disequilibrium were calculated with the Haploview software as described earlier.[40]

### Genotype-phenotype correlation

Genotype-phenotype correlations based on mutation profiles were assessed based on different demographic and clinical parameters. The quantitative clinical profiles of PCG patients at presentation were compared to the overall mutation spectra as well as common mutations in India (R368H) and Brazil (4340delG). All these clinical and demographic variables were subsequently fitted in a multivariate logistic regression model using the Akaike Information Criterion (AIC) with forward and backward elimination to estimate the adjusted odds ratio (OR). Statistical analysis was performed using the statistical software R (version 2.14.1; GNU General Public License). A value of p <0.05 was considered to be statistically significant.

## Results

### Demographic profile across PCG patients

The demographic profile of the Indian and Brazilian PCG patients are provided in Table 1. A lower age at onset and the presence of a positive family history of PCG was significantly associated with the presence of *CYP1B1* mutations among the Indian PCG cases but not among the Brazilians. The proportions of cases born out of consanguineous marriages in the mutation and non-mutation group were similar in both the Indian and Brazilian cohorts.

**Table 1:** Demographic profile of PCG patients in India and Brazil.

Demographic parameters	India (n = 301)			Brazil (n = 150)		
	*CYP1B1*(+)^a^ [n = 132]	*CYP1B1*(-)^b^ [n = 169]	p value	*CYP1B1*(+)^a^ [n = 66]	*CYP1B1*(-)^b^ [n = 84]	p value
Age at onset	1.51±1.25	2.42±3.34	0.003	3.76±6.34	4.14±5.21	0.687
Family history	16 (12.1%)	7 (4.2%)	0.014	29 (43.9%)	27 (32.1%)	0.174
Consanguinity	64 (48.5%)	65 (38.7%)	0.193	13 (19.7%)	11 (13.1%)	0.370

^a^ *CYP1B1*(+): Patients harboring *CYP1B1* mutation;

^b^ *CYP1B1*(-): Patients devoid of *CYP1B1* mutation

### Mutation spectrum of *CYP1B1* across PCG cases

The overall frequencies of *CYP1B1* mutations were similar between the Indian and Brazilian patients (Table 2). However, significantly higher frequencies of homozygous mutations were observed in the Indian cohort (p = 0.024) and compound heterozygous mutations the Brazilian cohort (p = 0.012), respectively. The mutation spectrum of *CYP1B1* was slightly different between the two populations and relatively higher number of mutations were observed among the Indian patients (n = 39) compared to the Brazilians (n = 17), which could be due to the variation in their sample sizes. Only six mutations were shared across these populations (Table 3). A detailed list of all the observed *CYP1B1* mutations in the Indian and Brazilian cohorts is provided as a Supplementary Table (S1 Table). Both these populations exhibited significant allelic heterogeneity with the R368H and the 4340delG being the prevalent mutations in India and Brazil, respectively. Interestingly, the prevalent mutation in Brazil (4340delG) was not observed in the Indian population and the R368H was observed only in three Brazilian patients in homozygous and compound heterozygous forms.

**Table 2:** Distribution of *CYP1B1* mutations in India and Brazil.

Mutation Profile	Type of mutation	India n, (%, [95%CI])	Brazil n, (%, [95%CI])	P value
Cases with *CYP1B1* Mutation	Total number of cases with any *CYP1B1* mutations	132 (43.85%, [95%CI, 38.36%-49.50%])	66 (44.00%, [95%CI, 36.30%-51.99%])	0.999
	Homozygous mutations	73 (24.25%, [95%CI, 19.75%-29.39%])	25 (16.66%, [95%CI, 11.55%-23.45%])	0.024^a^
	Heterozygous mutations	41 (13.62%, [95%CI, 10.20%-17.95%])	22 (14.66%, [95%CI, 9.88%-21.21%])	0.748
	Compound heterozygous mutations	18 (5.98%, [95%CI, 3.81%-9.25%])	19 (12.66%, [95%CI, 8.26%-18.94%])	0.012^a^
Cases without any *CYP1B1* mutation	-	169 (56.15%, [95%CI, 50.49%-61.64%])	84 (56.00%, [95%CI, 48.00–69.61%])	0.999

^a^Statistically significant (p<0.05)

**Table 3:** Distribution of shared *CYP1B1* mutations in the Indian and Brazilian cohorts.

Mutation	Frequency of the mutant allele		P value
	India, n (% [95% CI])	Brazil, n (% [95% CI])	
R368H	97 (16.11%, [13.29%-19.26%])	3 (1.00%, [0.34%-2.89%])	<0.0001
P437L	6 (0.99%, [0.45%-2.15%])	4 (1.33%, [0.52%-3.37%])	0.738
A443G	1 (0.17%, [0.03%-0.93%])	4 (1.33%, [0.52%-3.37%])	0.044
S476P	1 (0.17%, [0.03%-0.93%])	1 (0.33%, [0.06%-1.86%])	0.554
8037_8046dup10	2 (0.33%, [0.09%-1.20%])	14 (4.66%, [2.80%-7.67%])	<0.0001
8214_8215delAG	2 (0.33%, [0.09%-1.20%])	3 (1.00%, [0.34%-2.89%])	0.339

### *CYP1B1* haplotypes in India and Brazil

In order to assess the haplotype backgrounds of the observed *CYP1B1* mutations, six intragenic SNPs in the promoter (rs2617266), exon 2 (rs10012, rs1056827) and exon 3 (rs1056836, rs1056837, rs1800440) of *CYP1B1* were used to generate haplotypes in PCG cases and controls. It was observed that the mutation spectrum of *CYP1B1* was largely distributed across the four major haplotypes (>5%) in these two populations. As was seen earlier, the C-C-G-G-T-A was the risk haplotype while the T-G-T-C-C-A haplotype was protective in these populations.[45,48,50] The haplotype frequencies between the Indian and Brazilian PCG cases with and without *CYP1B1* mutations and the normal controls were not significantly different. As was noted earlier, the majority of the mutations clustered on the background of the C-C-G-G-T-A haplotype in both these populations.[40]

### Genotype-phenotype correlation of Indian and Brazilian PCG patients

The clinical profiles of PCG patients harboring *CYP1B1* mutations were compared to those without any mutations in both the cohorts under a logistic regression model. The demographic variables like gender, family history of the disease and history of consanguinity were treated as binary variables, while the presenting IOP and the presenting corneal diameter were analyzed as continuous variables. Although other measures like age at onset and cup to disk ratios were also recorded (wherever possible), they were not considered for further analysis due to their subjective determination across both the cohorts. Being an autosomal recessive disease, the analysis was repeated for the same clinical and demographic variables with respect to the subjects harboring homozygous mutations compared to those with heterozygous mutations.

Logistic regression revealed that neither gender, nor history of consanguinity conferred any susceptibility to *CYP1B1* mutations in the Indian and Brazilian cohorts. A similar phenomenon was observed with respect to the presenting IOP and CD in patients harboring any *CYP1B1* mutation in either of these cohorts (Table 4). Re-analysis of the data with respect to the carriers of homozygous mutations, did not provide any additional risk for any of these variables compared to those harboring heterozygous mutations. The data was also sub-classified with respect to the prevalent mutation seen among the Indians (R368H) and Brazilians (4340delG) compared to the other mutations with respect to these clinical and demographic variables. Interestingly, these mutations did not confer any additional susceptibility with respect to all the parameters across these two cohorts (Table 5).

**Table 4:** Logistic regression showing the adjusted odds ratios for the binary and continuous variables for different mutation categories in the Indian and Brazilian cohorts.

Variables	Parameters	"All *CYP1B1* mutations" versus "No *CYP1B1* mutations"				All "Homozygous *CYP1B1* mutations" versus "Heterozygous *CYP1B1* mutations"			
		India	India	Brazil	Brazil	India	India	Brazil	Brazil
		Adjusted OR (95%CI)	P value	Adjusted OR (95%CI)	P value	Adjusted OR (95%CI)	P value	Adjusted OR (95%CI)	P value
Binary	Gender (Male)	0.55 (0.25–1.99)	0.131	0.42 (0.13–1.41)	0.160	0.35 (0.10–1.28)	0.112	1.96 (0.28–13.83)	0.502
	History of consanguinity	0.84 (0.39–1.81)	0.650	0.34 (0.02–5.58)	0.448	0.40 (0.10–1.52)	0.178	0.24 (0.03–2.28)	0.215
Continuous	IOP	1.02 (0.96–1.09)	0.481	1.05 (0.95–1.15)	0.284	0.98 (0.86–1.11)	0.751	0.93 (0.79–1.10)	0.395
	CD	0.73 (0.50–1.06)	0.099	1.36 (0.65–2.82)	0.416	1.10 (0.59–2.07)	0.755	0.67 (0.17–2.69)	0.573

**Table 5:** Logistic regression showing the adjusted odds ratios for the binary and continuous variables with the prevalent *CYP1B1* mutation in the Indian and Brazilian cohorts.

Variables	Parameters	"The prevalent mutation" versus "Other *CYP1B1* mutations"			
		India (R368H)	India (R368H)	Brazil (4340delG)	Brazil (4340delG)
		Adjusted OR (95%CI)	P value	Adjusted OR (95%CI)	P value
Binary	Gender (Male)	1.96 (0.50–7.62)	0.334	1.45 (0.17–12.56)	0.734
	History of consanguinity	3.24 (0.88–11.95)	0.078	0.98 (0.06–14.8)	0.986
Continuous	IOP	0.95 (0.83–1.08)	0.410	1.06 (0.90–1.26)	0.471
	CD	1.84 (0.98–3.46)	0.059	0.96 (0.22–4.25)	0.957

## Discussion

The involvement of *CYP1B1* mutations with PCG has been widely demonstrated across different populations worldwide.[10,11,17] Despite the identification of over 100 *CYP1B1* mutations in PCG, thorough genotype-phenotype correlation studies are very scant in the literature.[42–47] Majority of the studies were limited in number of PCG cases as a whole and those harboring mutations, which often precluded from undertaking this efforts.[35,43,44,47] In order to address this lacunae, a comprehensive study was planned on a large sample (n = 901), comprising 451 PCG cases and 450 controls representing cohorts from India and Brazil. These two ethnically diverse and geographically distant populations represented a diverse mutation spectrum in *CYP1B1* along with variable clinical manifestation that helped in undertaking genotype-phenotype correlation.

In our effort to understand if any of the demographic or the presenting clinical parameters had a bearing on the susceptibility to mutations, all the PCG cases that either harbored or were devoid of any *CYP1B1* mutations in these two populations were analyzed. As evident from Table 1, a lower age at onset and a positive family history of the disease was associated with the occurrence of any *CYP1B1* mutation in the Indian cohort. An early age at onset was also observed in *CYP1B1*-associated PCG patients of South Korean and Lebanese origin.[33,47] Conversely, this was not observed in the Brazilian patients who had a relatively higher age at onset and an equal distribution of cases with positive family history in the mutation and non-mutation groups. While consanguinity per se, was not associated with the *CYP1B1* mutations in either of the cohorts, Indian PCG cases harboring mutations (p<0.0001) and those devoid of mutations (p<0.0001) exhibited a significantly higher proportion of consanguinity compared to the Brazilians.

As evident from Table 2, the mutation frequencies of *CYP1B1* in PCG were not grossly different between the Indian and Brazilian cohorts. While Indians had a slightly higher proportion of homozygous mutations (p = 0.024), so was for the Brazilian cases with the compound heterozygous mutations (p = 0.012). Allelic heterogeneity of *CYP1B1* was evident as 44 unique mutations were observed in the Indian (n = 33) and Brazilian (n = 11) patients. Only 6 mutations (8037_8046dup10, 8214_8215delAG, R368H, P437L, A443G and S476P) were shared between these two patient cohorts. Except for the R368H, the other shared mutations were observed in relatively lesser frequencies in the Indian patients (Table 3). It was seen that the allele frequencies of the prevalent mutations in a cohort was significantly different from the other cohort. For instance, the most prevalent Indian mutation R368H was observed only in 3 Brazilian patients (p<0.0001), but their common mutation 4340delG was not present in the Indian patients. Likewise, the second prevalent Brazilian mutation (8037_8046dup10) was observed in only one Indian patient with the homozygous mutant alleles (p<0.0001). A relatively larger number of *CYP1B1* mutations were observed in the background of all the 4 major haplotypes in the Indian cohort compared to the Brazilians, with some of them being observed on multiple haplotypes. Interestingly, the shared mutations in these two populations largely occurred on the same intragenic haplotypes providing further evidence for founder effects and population movements, as was demonstrated in our earlier study.[40]

Since the overall allele frequencies of the shared and unique mutations were not significantly different between the Indian and Brazilian patients, further genotype-phenotype correlations were initially based on the pooled data of all the observed mutations across these two cohorts to understand their implications on the demographic and clinical manifestations. However, the patients harboring the most prevalent mutations among the Indians (R368H) and the Brazilians (4340delG) were also analyzed separately to get an overall insight with respect to their clinical manifestations.

As evident from the Tables 4 and 5, our data did not indicate any correlation of the genotype(s) with phenotype(s) based on logistic regression. The adjusted odds ratios for the binary and continuous variables did not provide any additional susceptibility for any type of *CYP1B1* mutations in the Indian and Brazilian cohorts. The analysis with homozygous mutations only, did not provide any additional insights compared to the patients harboring a single copy of the mutant allele (heterozygous) in these two populations. This lack of correlation was consistent for the prevalent mutations in the Brazilian and Indian cohorts. This data therefore demonstrate a consistent trend with respect to the susceptibility of *CYP1B1* mutations among the demographic and clinical parameters at onset and indicate that a mutation per se, would not confer any additional risk in terms of disease severity to the patients in either population. These observations are interesting with respect to the underlying involvement of *CYP1B1*, since it happens to be the only major gene mapped in PCG so far. This is in contrast to another study wherein, the clinical characteristics varied widely across three different *CYP1B1*-associated ethnic groups comprising the Muslim Arabs and Druze and the Ashkenazi Jews in an Israeli population.[47]

While the precise role of *CYP1B1* in PCG is yet unclear, some *in vitro* and *in vivo* studies have demonstrated its involvement in development based on the retinoic acid signalling and also in the 17b estradiol formation.[17, 51] It has also been suggested that mutations in *CYP1B1* affect the development of TM by the degradation of some endobiotic compound that is necessary for the development of filtering structures.[52,53] An earlier study on the histological sections of 6 PCG patients indicated that cases with specific mutations exhibited severe to moderate angle dysgenesis.[44] Unfortunately, many of these studies were limited by the vagaries of small sample size and the associations with respect to the clinical outcomes have not been replicated universally. Similarly, some studies also demonstrated the implication of gene mutations based on the disease severity [39,44,46,47,], but these associations were grossly inconsistent in other populations.[27,33]

In summary, the present data suggested a lack of genotype-phenotype correlation with respect to the demographic and clinical parameters at onset of the disease. This is consistent with studies from South Korea and Kuwait.[27,33] Compared to other studies, the present data is robust in terms of sample size and has uniformly captured several parameters to undertake this genotype phenotype correlation in these two ethnically diverse and geographically distant populations from India and Brazil. Thus, the data would have further implications for understanding the involvement of *CYP1B1* mutations in the progression of clinical traits assessed through long-term follow up across these two patient cohorts. This may also aid in disease management and prognosis.

## Supporting Information

S1 Table The overall distribution of *CYP1B1* mutations observed in the Indian and Brazilian cohort. (DOCX)
