# Supplementary material for: Genotype-Phenotype Correlations in CYP1B1-Associated Primary Congenital Glaucoma Patients Representing Two Large Cohorts from India and Brazil
Source: PLoS One. 2015 May 15;10(5):e0127147. doi: 10.1371/journal.pone.0127147 (PMC4433271; doi:10.1371/journal.pone.0127147)
Supplement: S1 Table — (DOCX) [file pone.0127147.s001.docx]

**S1 Table. The overall distribution of *CYP1B1* mutations observed in the Indian and Brazilian cohort**

| **Sl. No.** | **Genomic DNA position** | **Amino acid change** | **SIFT score** | **%Allele frequencies** | | **Status of variation** | **Population(s) where the mutation was observed** |
| --- | --- | --- | --- | --- | --- | --- | --- |
|  |  |  |  | **India** | **Brazil** |  |  |
| 1 | g. 3775 insA |  | - | 2 (0.33%) | - | Reported | India (26,42) |
| 2 | g.3860C>T | p.Q19X | - | - | 3 (1%) | Reported | Brazil (48,50), South Korea (33) |
| 3 | g.3905-3927del | p.H34fs | - | 2 (0.33%) | - | Reported | India (26) |
| 4 | g.3925delG | p.R41fs | - | 2 (0.33%) | - | Novel | Present Study |
| 5 | g.3972 C>T | p.A56V | 0.06 | 1 (0.16%) | - | Novel | Present Study |
| 6 | g.3976 G>A | p.W57X | - | - | 6 (2%) | Reported | Australia (31), US (37), Germany (41), Brazil (48) |
| 7 | g.3987 G>A | p.G61E | 0.00 | 9 (1.49%) | - | Reported | Kuwait (27), India (42), Lebanese (47), USA (43), Saudi Arabia (20, 39) |
| 8 | g.4046 T>A | p.Y81N | 0.00 | 8 (1.32%) | - | Reported | Germany (41) |
| 9 | g.4055 G>T | p.V84F | 0.00 | 1 (0.17%) | - | Novel | Present Study |
| 10 | g.4095 T>C | p.L97P | 0.00 | 1 (0.17%) | - | Novel | Present Study |
| 11 | g.4148 G>C | p.A115P | 0.02 | 2 (0.33%) | - | Reported | India (26), Pakistan (35) |
| 12 | g.4197 C>G | p.S131R | 0.00 | 1 (0.17%) | - | Novel | Present Study |
| 13 | g.4200 T>G | p.M132R | 0.00 | 4 (0.67%) | - | Reported | India (26) |
| 14 | g.4236 A>C | p.Q144P | 0.06 | 1 (0.17%) | - | Reported | India (26) |
| 15 | g.4340delG | p.A179fsX18 | - | - | 37 (12.33%) | Reported | Morocco (23), USA (43), Brazil (48,50) |
| 16 | g. 4347 T>C | p.L181P | 0.00 | 2 (0.33%) | - | Novel | Present Study |
| 17 | g.4383 C>T | p.P193L | 0.01 | 2 (0.33%) | - | Reported | India (26,42) |
| 18 | g.4421_4423del | p.205delS | - | 2 (0.33%) | - | Novel | Present Study |
| 19 | g.4490G>A | p.E229K | 0.10 | 18 (2.98%) | - | Reported | Present Study |
| 20 | g.4520 A>C | p.S239R | 0.00 | 6 (0.99%) | - | Reported | India (26) |
| 21 | g.4523delC | p.L240fs | - | - | 1 (0.33%) | Novel | Present Study |
| 22 | g.4635delT | p.L277fsX1 | - | - | 6 (2%) | Reported | Brazil (50) |
| 23 | g.4641A>C | p.H279P | 0.00 | 3 (0.50%) | - | Novel | Present Study |
| 24 | g.4645C>A | p.C280X | - | 1 (0.17%) | - | Reported | Japan (21), Kuwait (27) |
| 25 | g.4793 G>A | p.A330T | 0.00 | 1 (0.17%) | - | Novel | Present Study |
| 26 | g.7900 C>T | p.R355X | - | 3 (0.50%) | - | Reported | US (37), Turkey (41) |
| 27 | g.7900_7901delCG | p.R355fs | - | 4 (0.67%) | - | Reported | India (26) |
| 28 | g.7917 G>A | p.L360L | - | 1 (0.17%) | - | Novel | Present Study |
| 29 | g.7901_7913del | p.R355fsX69 | - | - | 12 (4%) | Reported | France (24), Saudi Arabia (39), USA (41) |
| 30 | g.7940G>A | p.R368H | 0.00 | 97 (16.06%) | 3 (1%) | Reported | Saudi Arabia (8), Iran (19), India (26, 42), Turkey (30), Australia (31), USA (41) |
| 31 | g. 7949 G>T | p.C371F | 0.06 | 1 (0.17%) | - | Novel | Present Study |
| 32 | g.7970 T>A | p.L378Q | 0.00 | - | 2 (0.67%) | Reported | Brazil (50) |
| 33 | g.7996 G>A | p.E387K | 0.00 | - | 4 (1.33%) | Reported | Roma Gypsy (7), France (24),  Australia (31), US (49), Brazil (49, 50) |
| 34 | g.8005C>T | p.R390C | 0.00 | 19 (3.15%) | - | Reported | India (26) |
| 35 | g.8005 C>A | p.R390S | 0.00 | 2 (0.33%) | - | Reported | Iran (29), China (32), South Korea (33), Pakistan (35), Saudi (39), USA (43) |
| 36 | g.8006 G>A | p.R390H | 0.00 | 9 (1.49%) | - | Reported | China (32), South Korea (33),  Pakistan (35) |
| 37 | g.8035 C>T | p.P400S | 0.00 | - | 1 (0.33%) | Reported | Spain (53) |
| 38 | g.8037_8046dup | p.T404fsX30 | - | 2 (0.33%) | 14 (4.66%) | Reported | France (24), India (26), Turkey (30),  USA (41), Brazil (50) |
| 39 | g.8147C>T | p.P437L | 0.00 | 6 (0.99%) | 4 (1.33%) | Reported | India (26), Saudi Arabia (39), Brazil (50), Spain (53) |
| 40 | g.8148del 5bp | p.P437fs | - | 2 (0.33%) | - | Novel | Present Study |
| 41 | g.8162C>T | p.P442L | 0.02 | 1 (0.17%) | - | Novel | Present Study |
| 42 | g.8165 C>G | p.A443G | 0.00 | 1 (0.17%) | 4 (1.33%) | Reported | Saudi Arabia (39), Brazil (50) |
| 43 | g.8168 G>A | p.R444Q | 0.00 | - | 1 (0.33%) | Reported | Japan (21), Lebanon (48), Spain (53) |
| 44 | g.8182delG | p.D449fs | - | - | 7 (2.33%) | Reported | Brazil (50) |
| 45 | g.8214_8215delAG | p.V460fs | - | 2 (0.33%) | 3 (1%) | Reported | Brazil (50) |
| 46 | g.8227 T>C | p.S464P | 0.01 | 1 (0.17%) | - | Novel | Present Study |
| 47 | g.8234 G>A | p.G466D | 0.01 | 2 (0.33%) | - | Reported | India (26), Saudi Arabia (39) |
| 48 | g.8242 C>T | p.R469W | 0.00 | 1 (0.17%) | - | Reported | USA (41), Brazil (50) |
| 49 | g. 8263 T>C/  g.8264 C>A | p.S476P/  p.S476Y | 0.01 | 1 (0.17%) | 1 (0.33%) | Novel | Present Study |
| 50 | g. 8393 A>G | p.N519S | 0.82 | 1 (0.17%) | - | Novel | Present Study |
